# Introducing the *Journal of the Medical Library Association*'s policy on the use of generative artificial intelligence in submissions

**DOI:** 10.5195/jmla.2023.1826

**Published:** 2023-10-02

**Authors:** Jill T. Boruff, Michelle Kraft, Alexander J. Carroll

**Affiliations:** 1 jill.boruff@mcgill.ca, Co-Lead Editor, *Journal of the Medical Library Association*, Associate Librarian, Schulich Library of Physical Sciences, Life Sciences, and Engineering, McGill University, Montreal, QC, Canada.; 2 kraftm@ccf.org, Co-Lead Editor, *Journal of the Medical Library Association*, Medical Library Director, Cleveland Clinic Foundation, Cleveland, OH, United States.; 3 alexander.j.carroll@vanderbilt.edu, Associate Director, Stevenson Science and Engineering Library, Vanderbilt University, Nashville, TN, United States.

## Abstract

With the arrival of ChatGPT, the academic community has expressed concerns about how generative artificial intelligence will be used by students and researchers alike. After consulting policies from other journals and discussing among the editorial team, we have created a policy on the use of AI on submissions to *JMLA*. This editorial provides a brief background on these concerns and introduces our policy.

Artificial intelligence (AI) technologies and applications have the potential to enhance how librarians and researchers work with information. On November 30, 2022, OpenAI publicly launched ChatGPT, a free web application known as a “chatbot” using AI to interact with people and generate responses to their queries [1]. While chatbots have been employed prior, ChatGPT created waves due to the speed of its responses, the “human sounding” language it used in its responses, and its ability to adapt to subsequent questions. ChatGPT is a large language model (LLM) AI, a generative artificial intelligence that creates sentences using patterns of language structure in its database to generate paragraphs of text like that of a human writing the text. For librarians interested in learning more about the relevant terms and technology in this space, Lorcan Dempsey provides an excellent primer [2].

With the arrival of ChatGPT, concerns about academic integrity quickly surfaced. Professors discovered students submitting papers generated by ChatGPT [3], and researchers testing the AI's abilities discovered it can answer and pass exams in business and law as well as the United State Medical Licensing Exam (USMLE) [4]. ChatGPT also generates false, but very realistic, citations to academic papers, which our readers may have encountered while working with their patrons. Researchers have even published conversations about plagiarism with ChatGPT [5].

Scholarly publishers have likewise been affected by and monitoring the use of AI and ChatGPT within its ecosystem [6]. Early studies have found that ChatGPT is sophisticated enough to write scholarly articles for publication in academic journals [7]. Meanwhile, a recent article in Nature reported four articles published in peer reviewed journals listing an AI tool as co-author, raising concerns with publishing community over whether AI can be listed as an author and other concerns of transparency and responsibility [8]. However, the scientific community is far from consensus that this technology is entirely negative, with some researchers already advocating for seeking out opportunities for leveraging LLMs throughout the research life-cycle [9].

Journal editors, researchers, publishers, and the biomedical scientific community have begun to discuss the appropriate use of AI within research in order to establish policies on the use of AI for writing manuscripts and creation of images for publication. Among the issues currently being contested are: whether AI can be listed as an author [10]; the differences between using AI as part of the research methodology versus using it to write the manuscript [11]; and the use of AI to generate images and figures [12]. Interestingly, journal and publisher policies often settle on slightly different decisions at the margins of each issue, reflecting the divergence of opinion and lack of consensus in the space. We also anticipate that our colleagues' policies are likely to change as the AI landscape develops.

*JMLA* was confronted with the use of AI in a manuscript submission early in 2023. A manuscript on the future of AI in medical libraries was submitted for consideration by the journal. At first glance, it seemed like a typical submission. However, the citations were the first clue that something was different. Two *JMLA* articles were cited; and while these citations had the proper volume and issue numbers, the articles did not exist. Upon a closer read of the manuscript, the broad, generalized statements signaled that the paper was not written by a human. We used the AI-detection tool provided by ChatGPT to confirm that it was “highly likely” that the manuscript and citations were written by AI. However, these AI-detection tools are not without fault, with prominent tools such as Turnitin reporting false positive rates as high as 4% [13]. In fact, the AI-detection tool provided by Chat-GPT that we used in our process was itself sunset in July 20, 2023, due to concerns over its low rate of accuracy, less than 6 months after it was launched [14]. Reviewing this submission, and the inability to rely on tools such as these going forward, highlighted the exigent need for a policy on the use of AI.

After consulting policies from other journals and discussing among the editorial team, we have created a policy on the use of AI on submissions to *JMLA*. This policy (found on the *JMLA* site: https://jmla.mlanet.org/ojs/jmla/AIsubmissionpolicy) will certainly change as this technology and its uses evolve. While *JMLA*'s current policy calls for restrictions on the use of LLMs in the production of original content submitted to the journal, we recognize that these technologies have the potential to transform the discovery, processing, and synthesis of research. Indeed, it is the transformative potential of these tools that makes it imperative that they are utilized openly and transparently.

Equally important is for librarians to conduct research using and on the use of these tools for the profession to learn, adapt, and grow with the technology. As editors we encourage authors to submit articles that depict the integration of LLMs and generative artificial intelligence software into information science research and the professional practice of health science librarians. Projects and initiatives along these lines are already underway. The National Library of Medicine currently uses AI to select indexing terms in PubMed [15], while researchers at The Stanford Center for Research on Foundation Models (CRFM) and MosaicML released an AI trained to interpret biomedical language they originally called PubMedGPT but later renamed to BioMedLM [16]. Meanwhile, prompt engineering, the process of creating queries for an LLM-powered chatbot that will produce meaningful and relevant results, has been identified as a possible application of librarian expertise in search and retrieval [17].

The tasks highlighted in these examples (generating metadata, interpreting primary literature, and retrieving information) are core competencies of health information professionals. Twenty years ago, the ascendence of web-based information tools like Google and Wikipedia created angst among librarians that their skills and expertise would lose relevance for students and researchers. Yet over the course of the following two decades, the profession thoroughly integrated these tools into research and practice. Like Wikipedia and Google before it, we anticipate that LLMs, chatbots, and artificial intelligence software may become tools that librarians develop expertise in using to advance the research needs of their communities [18]. We look forward to the opportunity to review articles investigating the integration of AI and medical librarianship.
